# Rubisco kinetic diversity and effectiveness of CO_2_-concentrating mechanisms in aquatic photosynthetic organisms

**DOI:** 10.1093/plphys/kiag507

**Published:** 2026-07-16

**Authors:** Concepción Iñiguez, Sebastià Capó-Bauçà, Francisco J L Gordillo, Pablo Cobos, Pere Aguiló-Nicolau, Jeroni Galmés

**Affiliations:** Department of Ecology, Faculty of Sciences, University of Málaga, Málaga 29010, Spain; Research Group on Plant Biology Under Mediterranean Conditions, Universitat de les Illes Balears-INAGEA, Palma, Balearic Islands 07122, Spain; Department of Ecology, Faculty of Sciences, University of Málaga, Málaga 29010, Spain; Department of Ecology, Faculty of Sciences, University of Málaga, Málaga 29010, Spain; Research Group on Plant Biology Under Mediterranean Conditions, Universitat de les Illes Balears-INAGEA, Palma, Balearic Islands 07122, Spain; Research Group on Plant Biology Under Mediterranean Conditions, Universitat de les Illes Balears-INAGEA, Palma, Balearic Islands 07122, Spain

## Abstract

Aquatic photoautotrophs experience strong physicochemical constraints on inorganic carbon acquisition due to low CO_2_ and O_2_ diffusion in water producing a strong reduction in the gas conductance. These limitations have driven the widespread evolution of CO_2_-concentrating mechanisms (CCMs), which enhance CO_2_ availability around Rubisco and mitigate its catalytic inefficiencies.

A compilation of the Rubisco kinetics and apparent photosynthetic CO_2_ affinities (1/K_m CO2_) for a wide range of taxa shows that, despite the structural diversity of CCMs, there is an evolutive convergence among cyanobacteria, diatoms, haptophytes, and some rhodophytes and chlorophytes with similarly high 1/K_m CO2_, therefore operating almost CO_2_-saturated photosynthesis under ambient conditions. Notably, none of the compiled species achieves CO_2_ saturation solely through Rubisco kinetics, underscoring the central role of CCMs in aquatic carbon fixation.

CCM effectiveness (the ratio between Rubisco CO_2_ semi-saturation constant under 21% O_2_ and apparent photosynthetic semi-saturation constant for CO_2_, K_c_^air^/K_m CO2_) varies widely across the compiled species but is consistently higher in organisms possessing Rubisco-containing microcompartments, although pyrenoids are not strictly required for concentrating CO_2_ around Rubisco above ambient levels. Furthermore, an inverse relationship between Rubisco carboxylation efficiency and 1/K_m CO2_ supports a divergent CCM-Rubisco coevolutionary trajectory compared with terrestrial plants, likely influenced by intracellular photosynthetic O_2_ accumulation under submerged conditions.

The present review synthesizes current knowledge on CCM diversity and its regulation under future global change scenarios but also reanalyzes CCM effectiveness and its co-evolution with Rubisco kinetic traits across aquatic oxygenic phototrophs, highlighting major knowledge gaps to be filled by future research.

## Introduction

Aquatic photosynthetic organisms inhabit environments where the physicochemical constraints on inorganic carbon acquisition differ markedly from those experienced by terrestrial plants. In water at 25 °C, the diffusion of CO_2_ and O_2_ is ∼10,000-fold slower than in air (Zeebe 2011; Somerville and Proctor 2013), which provokes a substantial increase in the resistance to gas transport through the boundary layers surrounding organisms, especially in areas of low turbulence. This leads to a reduction in the intracellular CO_2_ levels under light conditions well below the values for air-equilibrated waters, limiting Rubisco carbon fixation. Moreover, the slow diffusion of gases in aquatic environments can also lead to the accumulation of photosynthetically produced O_2_ within cells and tissues. The resulting increase in the local O_2_ ratio around Rubisco may favor RuBP oxygenation, thus increasing photorespiration (Mass et al. 2010).

In marine ecosystems, stable pH and alkalinity conditions maintain CO_2_ as a minor fraction of the dissolved inorganic carbon (DIC) pool under air-equilibrium conditions (eg 0.7% at 15 °C and pH ∼8.1), and its salinity further reduces gas solubility, contributing to a strong and permanent CO_2_ limitation for photosynthesis. In freshwater environments, there is an enormous variability in alkalinity, which means that air-equilibrium [CO_2_] can vary between 100% of DIC in acid sites to <0.2% in places with a high alkalinity. However, on average, annual CO_2_ levels are 20 to 46 times lower than HCO_3_^−^ in rivers and lakes over the world, and dissolved CO_2_ can become depleted below air equilibrium in many freshwater ecosystems during periods of high photosynthetic activity (Maberly and Gontero 2017).

Such limitations have driven the polyphyletic evolution of CO_2_-concentrating mechanisms (CCMs) in most aquatic photosynthetic organisms, which allow them to use the most abundant DIC form, bicarbonate, for photosynthesis, finally increasing the CO_2_ concentration near Rubisco active sites (Giordano et al. 2005). This mitigates the inherent Rubisco catalytic constraints, such as the competitive RuBP oxygenation and the relatively low affinity for CO_2_, thereby allowing Rubisco to operate closer to its carboxylation potential and reducing the energetic and carbon cost associated to photorespiration (Iñiguez et al. 2020).

Aquatic CCMs encompass a broad range of biophysical components, including extracellular or intracellular carbonic anhydrases, active HCO_3_^−^/CO_2_ transport systems, and, in many species, the compartmentalization of Rubisco into specialized microstructures such as pyrenoids or carboxysomes (Beer and Beardall 2025). In addition, some submersed freshwater angiosperms have shown a relevant contribution of C_4_-like and CAM metabolism in concentrating CO_2_ around Rubisco active sites in combination with biophysical mechanisms (Jiang et al. 2026), although evidence for these biochemical mechanisms in algae (Reiskind and Bowes 1991; Reinfelder et al. 2000) is reduced and still debated (Beer and Beardall 2025). This structural and mechanistic diversity in aquatic CCMs could be translated into differences in the potential effectiveness to concentrate CO_2_ around Rubisco in response to either permanent (marine) or temporal (freshwater) CO_2_ limitation in their environment, but this has been poorly explored up to date (but see Clement et al. 2017 and Capó-Bauçà et al. 2022).

The presence and effectiveness of CCMs must, in turn, have exerted a strong selective pressure on the evolution of Rubisco kinetic properties of photosynthetic aquatic organisms. When a CCM elevates stromal or microcompartmental CO_2_ concentrations, the enzyme is partially relieved from the need for a high CO_2_ affinity (Yeoh et al. 1981). Therefore, a common characteristic among CCM expressing organisms is to have a higher Rubisco semi-saturation constant for CO_2_ (K_c_) relative to other phylogenetically related organisms not expressing CCMs. In terrestrial plants, this reduction in enzymatic CO_2_ affinity observed in those organisms with biochemical CCMs (C_4_ and CAM metabolisms) appears to be linked to higher carboxylation turnover rates (k_cat_^c^; Sharwood et al. 2016). This pattern of evolutive kinetic adjustment, previously described as a “canonical” trade-off between carboxylation velocity and substrate affinity (Tcherkez et al. 2006), has not been observed in most aquatic photoautotrophs (Young et al. 2016; Heureux et al. 2017; Iñiguez et al. 2019, 2020; Goudet et al. 2020; Capó-Bauçà et al. 2022, 2023a, 2023b). Instead, in many of these species, a lower carboxylation efficiency (k_cat_^c^/K_c_) was accompanied by a lower oxygenation efficiency (k_cat_^o^/K_o_), maintaining similar values of the specificity for CO_2_ over O_2_ (S_c/o_) than non-CCM relative species (Capó-Bauçà et al. 2022, 2023a, 2023b). Hence, other “canonical” trade-offs previously described between either k_cat_^c^/K_c_ or k_cat_^c^ and S_c/o_ (Tcherkez et al. 2006) were also weakened or, in some cases, even disappeared when comparing aquatic phylogenetically diverse groups. Whether this evolutionary pattern might have an advantage in aquatic ecosystems is still under investigation, although it might be related to the higher resistance to lose O_2_ out of the cell that occurs during submersed photosynthesis.

The present review synthesizes recent advances on the ecophysiology and effectiveness of CCMs and Rubisco kinetic diversity across aquatic oxygenic photoautotrophs. By integrating evidence from cyanobacteria, green and non-green micro and macroalgae, seagrasses, and aquatic angiosperms, we highlight convergent patterns across phylogenetically distant lineages that reveal common environmental pressures driving the coevolution between Rubisco kinetic traits and CCM effectiveness.

### Evolutionary trajectories of Rubisco kinetics in aquatic organisms

The photosynthetic carbon fixation rate depends on the intracellular CO_2_/O_2_ ratio, as well as the concentration of Rubisco active sites and the catalytic traits of the enzyme (Galmés et al. 2014). Rubisco presents important catalytic inefficiencies related to its dual function as carboxylase and oxygenase. The Rubisco specificity factor (S_c/o_) gives the number of carboxylations relative to oxygenations for any given concentration of CO_2_ and O_2_. In turn, S_c/o_ depends on 4 kinetic parameters defining the catalytic performance of the enzyme:


$$S_{c/o}=({k_{\mathrm{cat}}}^{\;c}/K_{c})/({k_{\mathrm{cat}}}^{\;o}/K_{o}),$$

where k_cat_^c^ and k_cat_^o^ are, respectively, the catalytic turnover rate for the carboxylase and the oxygenase reactions, which means the maximum number of moles of CO_2_ or O_2_ fixed per catalytic site and second, while K_c_ and K_o_ are the semi-saturation constants for CO_2_ and O_2_ and represent the inverse of the affinity for CO_2_ and O_2_, respectively. Therefore, the specificity factor can be defined as the ratio between the carboxylation efficiency (k_cat_^c^/K_c_) and the oxygenation efficiency (k_cat_^o^/K_o_).

The different phylogenetic groups inhabiting aquatic environments possess diverse Rubisco forms, with contrasting Rubisco kinetic parameters. Almost all aquatic oxygenic photoautotrophs express Rubisco form I, specifically the subtypes IA, IB or ID, with an hexadecameric L_8_S_8_ complex (Iñiguez et al. 2020). Eukaryotes only possess Form IB (green plastic clade) or form ID (red plastic clade), except for peridinin-containing dinoflagellates, in which form I Rubisco was replaced by a nucleus-encoded, single subunit form II Rubisco acquired by horizontal gene transfer from a proteobacterium (Morse et al. 1995; Raven et al. 2020). Prokaryotic oxygenic photosynthetic organisms (Cyanobacteria) possess either form IA or IB Rubiscos, although recent metagenomics analyses have revealed that certain lineages adapted to marine oxygen-deficient zones also encode the more primitive form II Rubisco, which is expressed under low-oxygen and low-light conditions (Jaffe et al. 2024). Beyond differences in the amino-acid sequence, Rubisco forms also vary in quaternary organization, assembly requirements, and activation mechanisms, leading to the large variability in Rubisco kinetic traits previously acknowledged (Iñiguez et al. 2020). However, the natural landscape of Rubisco kinetic diversity remains poorly explored due to the historical focus on land plants, albeit recent studies on Rubisco kinetics in previously underrepresented groups of aquatic photosynthetic organisms (Goudet et al. 2020; Capó-Bauçà et al. 2022, 2023a, 2023b; Aguiló-Nicolau et al. 2023, 2025; Steensma et al. 2025) can help to define the broader natural limits of Rubisco performance.

**Figure 1:**
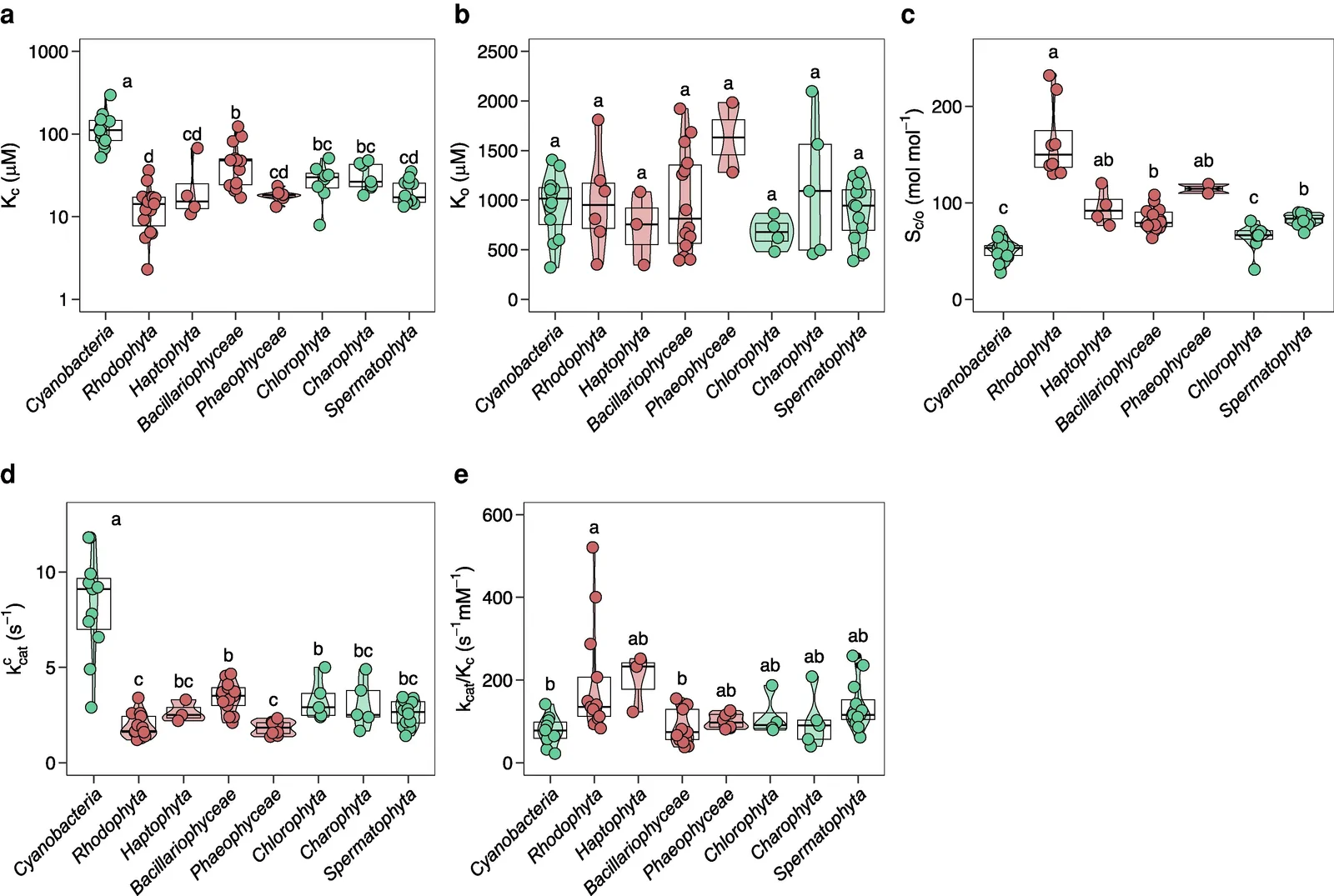
Rubisco kinetic parameters across aquatic oxygenic photosynthetic lineages. a-e) Violin plots show the distribution of the main Rubisco catalytic parameters compiled from the literature and corrected for methodological differences following Iñiguez et al. (2021) and normalized at 25 °C following the thermal equations described in Galmés et al. (2016) for 103 species belonging to major aquatic oxygenic photosynthetic lineages (Supplementary Dataset S1): (a) semi-saturation constant for CO_2_ (*K*_c_, µM; in log scale), (b) semi-saturation constant for O_2_ (*K*_o_, µM), (c) carboxylation turnover rate (*k*_cat_^c^, s^−1^), (d) specificity factor for CO_2_ over O_2_ (*S*_c/o_, mol mol^−1^), and (e) carboxylation efficiency (*k*_cat_^c^/*K*_c_, s^−1^ mM^−1^). Embedded boxplots indicate the median (central line), interquartile range (25th−75th percentiles; box), and whiskers represent the data range excluding outliers. Points represent individual species values. Colors indicate the green and red lineage (Rubisco form IA/IB and ID, respectively). Different letters indicate significant differences among phylogenetic groups (Kruskal–Wallis test followed by pairwise comparisons with Bonferroni correction; *P* < 0.05).

With this aim, we made a compilation and correction for methodological differences (Iñiguez et al. 2021) of the main Rubisco kinetic parameters reported up to date for 103 species of aquatic photosynthetic organisms, revealing significant differences among the main aquatic phylogenetic groups (Cyanobacteria, Chlorophyta, Charophyta, Spermatophyta, Rhodophyta, Ochrophyta, and Haptophyta). In particular, the lowest affinities for CO_2_ (highest K_c_) were found in Cyanobacteria followed by diatoms (Bacillariophyceae, Ochrophyta), whereas the highest affinities for CO_2_ (lowest K_c_) were observed in submersed spermatophytes, Phaeophyceae (Ochrophyta), Haptophyta, and Rhodophyta (Fig. 1a). On the contrary, the highest k_cat_^c^ values were obtained in Cyanobacteria, followed by diatoms and Chlorophyta, while the lowest values were obtained in Rhodophyta and Phaeophyceae (Fig. 1d). As a result, the lowest carboxylation efficiencies were observed in Cyanobacteria and diatoms and the highest carboxylation efficiencies in Rhodophyta (Fig. 1e). No significant differences in K_o_ were detected among groups, likely reflecting the large intrinsic variability associated with its indirect estimation (Iñiguez et al. 2021; Fig. 1b). Finally, the highest S_c/o_ values were found in Rhodophyta, followed by Phaeophyceae, whereas the lowest values were obtained for Cyanobacteria and Chlorophyta (Fig. 1c).

Examples of species with extreme Rubisco kinetics include the thermoacidophilic rhodophytes *Galdieria sulphuraria*, which exhibits the highest Rubisco affinity for CO_2_ (K_c_ = 2.3 µM), the lowest k_cat_^c^ (1.2 s^−1^), and the highest k_cat_^c^/K_c_ (521 s^−1^ mM^−1^) ever reported, and *Galdieria partita*, that possesses the highest S_c/o_ (232 mol mol^−1^). In contrast, the primitive cyanobacterium *Gloeobacter violaceus* shows one of the highest k_cat_^c^ values (11.8 s^−1^). At the opposite extreme, the marine α-cyanobacterium *Prochlorococcus marinus* MIT9313 exhibits the highest K_c_ (296 µM) together with the lowest k_cat_^c^/K_c_ (22 s^−1^ mM^−1^), a pattern similarly observed (albeit to a lesser degree) in the charophyte *Coleochaete scutata* and in the Arctic diatoms *Thalassiosira nordenskioeldii* and *Thalassiosira antarctica*, all characterized by similarly low carboxylation efficiencies (Supplementary Dataset S1). The only peridinin-containing dinoflagellate analyzed to date, *Amphidinium carterae*, which possess Rubisco form II, showed the lowest S_c/o_ among aquatic oxygenic photoautotrophs (22 mol mol^−1^, recalculated at 25 °C by using the thermal dependencies reported by Galmés et al. 2016), followed by form IB Rubiscos from *Chlorella pyrenoidosa* and *Nostoc* sp. PCC7120 (31 and 28 mol mol^−1^, respectively).

There is an extensive discussion regarding the main drivers of Rubisco kinetic evolution provoking these remarkable differences. The relative importance of structurally and chemically constrained kinetic trade-offs versus phylogenetic constraints in Rubisco kinetic evolution have been acknowledged and debated (Cummins et al. 2018; Tcherkez et al. 2018; Bouvier et al. 2021; Tcherkez and Farquhar 2021). The intracellular CO_2_/O_2_ microenvironment has also been proposed as another relevant driver in Rubisco evolution (Tcherkez et al. 2018). Recent studies supported the importance of the environment and CCM effectiveness on Rubisco kinetic adaptation in aquatic ecosystems, even over phylogenetic and trade-off constraints, revealing convergent evolution in phylogenetically distant species (Iñiguez et al. 2020; Capó-Bauçà et al. 2022, 2023a).

### Mechanistic diversity in aquatic CCMs

The intracellular CO_2_/O_2_ ratio around Rubisco active sites is mainly determined by environmental factors (eg higher temperature decrease gas solubility and the CO_2_/O_2_ ratio), morpho-anatomical characteristics determining the diffusive resistance to CO_2_, and physiological mechanisms allowing CO_2_ to be concentrated.

Aquatic CCMs are functionally convergent but structurally diverse. In Cyanobacteria, CCMs are universally present and are based on a set of bicarbonate transporters, CO_2_ uptake complexes, and the physical encapsulation of carbonic anhydrase (CA) and Rubisco in icosahedral proteinaceous microcompartments, the carboxysomes. Active uptake of bicarbonate via multiple transport systems leads to the accumulation of high cytosolic HCO_3_^−^ concentrations, while CO_2_ uptake complexes associated with thylakoid membranes can further contribute to increasing cytosolic HCO_3_^−^ pool (Rae et al. 2013). Within the carboxysome, CA-mediated dehydration of HCO_3_^−^ generates elevated CO_2_ levels around Rubisco, while the proteinaceous shell potentially restricts CO_2_ leakage. There are 2 evolutive lineages, α-carboxysomes encapsulating form IA Rubisco, which are present in cyanobacteria dominating both open-ocean and freshwater ecosystems (Cabello-Yeves et al. 2022), and β-carboxysomes encapsulating form IB Rubisco, which are present in cyanobacteria mainly distributed in coastal/estuarine or freshwater/terrestrial ecosystems. Our data compilation reinforced the idea that, although both carboxysome types possess significant structural differences (Zhou et al. 2024), they achieve similar functional outcomes, as supported by similar apparent photosynthetic affinity for CO_2_ (Whitehead et al. 2014; see Supplementary Dataset S2). However, this conclusion must be taken with caution because data for α-cyanobacteria are still limited.

In eukaryotic algae, increased CO_2_ levels around Rubisco active sites can be achieved through a combination of membrane bicarbonate/CO_2_ transporters, periplasmic and cytosolic CAs, and the frequent appearance of 1 or more pyrenoids in the chloroplast, a phase-separated condensate characterized by the aggregation of Rubisco, CA, and linker proteins (Hopkinson et al. 2016; Long et al. 2024), functionally equivalent to prokaryotic carboxysomes. Pyrenoids are present in all algal phyla and many hornworts but are absent in vascular plants (Barrett et al. 2021). Most of our molecular, structural, and functional knowledge about pyrenoids is based primarily on work in the model chlorophyte *Chlamydomonas reindhartii* (reviewed by Catherall et al. 2025), although very recent efforts are expanding this knowledge into model diatoms and other algae (Oh et al. 2023; Barrett et al. 2024; Moromizato et al. 2024; Shimakawa et al. 2024). These studies revealed that pyrenoids are morphologically and structurally diverse among the different algal groups, with lineage-specific linker proteins and different structural components mediating Rubisco condensation, confirming that they evolved independently multiple times across algal lineages (Long et al. 2024). In *C. reindhartii*, pyrenoids are traversed by modified thylakoid tubules that deliver HCO_3_^−^ to luminal CAs, which, at the acidic luminal pH, produce CO_2_ that diffuses into the pyrenoid matrix where Rubisco is allocated. CO_2_ leakage is prevented by a starch sheath, which forms a diffusion barrier surrounding the pyrenoid (Catherall et al. 2025). Contrarily, in diatoms, pyrenoids are encapsulated by a proteinaceous shell that constrains CO_2_ diffusion and is essential for organelle integrity and efficient CO_2_ fixation (Nam et al. 2024; Shimakawa et al. 2024). In both chlorophytes and diatoms, bicarbonate is transported into the luminal thylakoid tubules by bestrophin-like proteins (BST).

Some green and non-green algae, as well as seagrasses and some freshwater angiosperms, operate effective CCMs without pyrenoids (Badger et al. 1998; Raven et al. 2014), although their molecular mechanisms are still largely unknown. For example, the thermoacidophilic red alga *Cyanidioschyzon merolae* likely operates a minimal pH-gradient-driven CCM in which CO_2_ passively diffuses into the cell and then is converted to bicarbonate in the cytosol by CAs. Bicarbonate is subsequently transported into the chloroplast via active transporters and transformed into CO_2_ near Rubisco active sites by chloroplastic CAs (Steensma et al. 2025). The difference in pH between the acidic external medium and the nearly neutral cytosol eliminates the need for an energy-consuming DIC uptake. The streptophyte alga *Chara braunii* operates a surface acidification-based CCM in which localized proton extrusion (most likely mediated by membrane-localized P-type ATPases) acidifies discrete regions of the cell surface, shifting carbonate equilibria toward higher pCO_2_, that passively diffuses into the cytosol (Heise et al. 2025). This effect is likely reinforced by external CAs and aquaporins that enhance CO_2_ permeability and that, together with bicarbonate transporters and internal CAs, might all be responsible for the final increase in CO_2_ around Rubisco active sites. Such acidification-based CCMs are likely favored in larger aquatic phototrophs because relatively thick diffusive boundary layers allow substantial deviations in pH and carbonate chemistry at the cell surface from those of the bulk medium. In contrast, this mechanism is expected to be less effective in small unicellular algae, where thinner boundary layers promote the rapid dissipation of proton and CO_2_ gradients (Flynn et al. 2012; Raven and Beardall 2016). In fact, acidification of localized regions of the periplasmic space driven by H^+^ extrusion pumps has been proposed as a primary DIC uptake mechanism in seagrasses and some seaweeds, as evidenced by the strong inhibition of photosynthesis observed following the use of pH buffers and external carbonic anhydrase inhibitors (Capó-Bauçà et al. 2022, 2023a). This mechanism may also operate in concert with direct bicarbonate acquisition via H ^+^ -HCO_3_^−^ symport, as demonstrated in the seagrass *Posidonia oceanica* (Rubio et al. 2017). Therefore, pyrenoids do not appear to be a prerequisite for effectively concentrating CO_2_ around Rubisco, although the tight packaging of Rubisco within a microcompartment permits inorganic carbon accumulation with limited CO_2_ leakage (Beer and Beardall 2025).

Beyond these biophysical mechanisms, some submerged aquatic angiosperms can additionally employ biochemical CCMs, showing C_4_-like and/or CAM metabolisms. These biochemical CCMs consist of an initial carboxylation step catalyzed by phosphoenolpyruvate carboxylase, which fixes HCO_3_^−^ into a C_4_-sugar that will be later decarboxylated in the proximity of Rubisco (Sage 2004). Evidence for the presence of these mechanisms in aquatic angiosperms has been reported in species such as *Hydrilla verticillata*, *Egeria densa*, and *Ottelia alismoides* (Magnin et al. 1997; Casati et al. 2000; Jiang et al. 2026), indicating that submerged angiosperms can integrate both biophysical and biochemical CCM components, allowing them to sustain photosynthesis under CO_2_ limitation.

### Is aquatic photosynthesis mostly saturated at ambient CO_2_ conditions?

**Figure 2:**
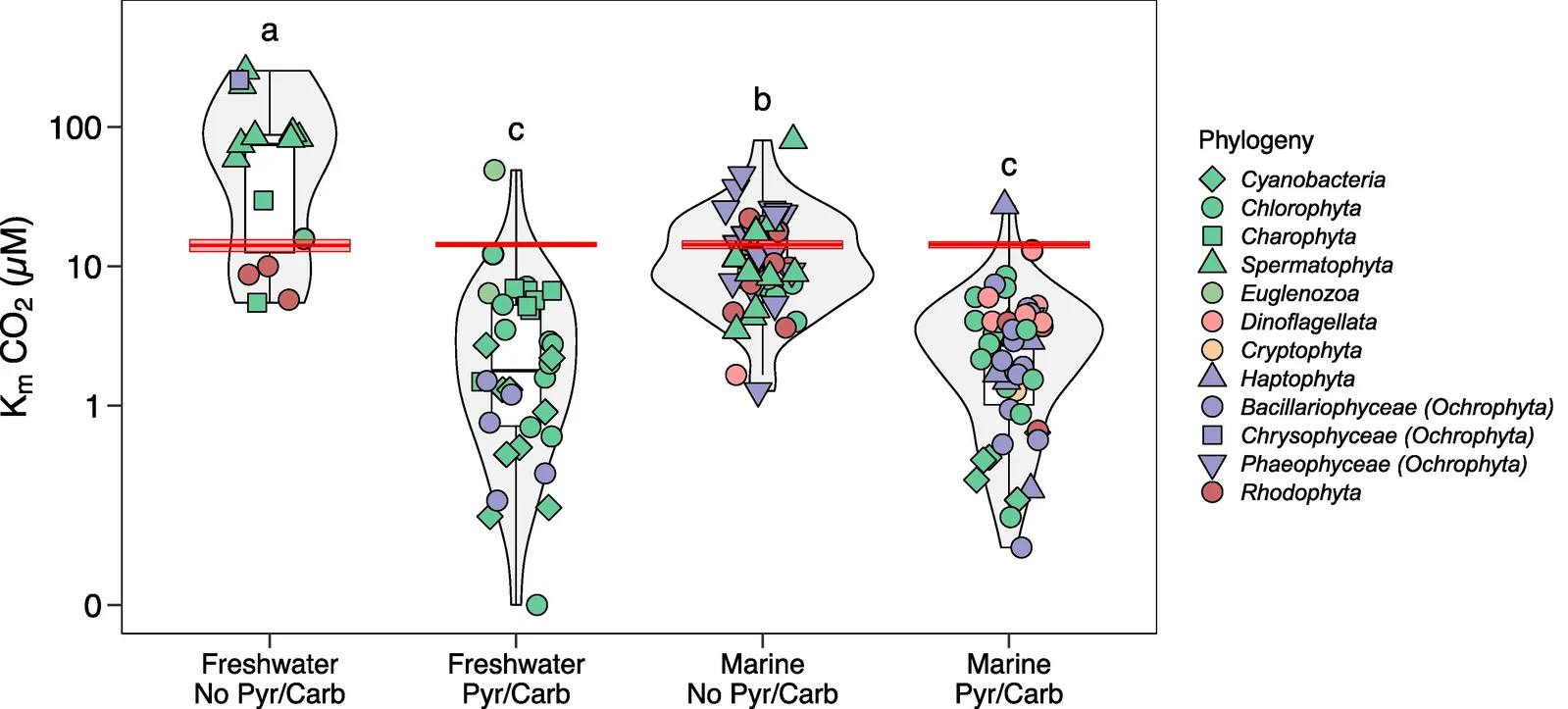
Apparent photosynthetic affinity for CO_2_ across aquatic oxygenic photoautotrophs. Violin plots show the distribution of the apparent photosynthetic semi-saturation constant for CO_2_ (K_m CO2_, µM; in log scale) compiled from the literature for 154 species belonging to major aquatic oxygenic photosynthetic lineages (Supplementary Dataset S2). Embedded boxplots indicate the median (central line), the interquartile range (25th–75th percentiles; box), and whiskers represent the data range excluding outliers. Points represent individual species, identified by their phylogenetic group. Data are grouped by habitat (freshwater vs marine) and by the presence/absence of a Rubisco-containing microcompartment (pyrenoid in eukaryotic algae or carboxysome in Cyanobacteria). Red horizontal lines indicate the expected range of dissolved CO_2_ concentrations under air-equilibrium conditions for the environmental temperatures and salinities at which the organisms were assayed. Different letters indicate significant differences among groups (Kruskal–Wallis test followed by pairwise comparisons with Bonferroni correction; *P* < 0.05). When the literature only reported the apparent photosynthetic semi-saturation constant for dissolved inorganic carbon (K_m DIC_), this value was converted to an equivalent K_m CO2_ by using the CO2SYS software, accounting for carbonate system speciation as a function of temperature, salinity and pH of each measurement. CO2SYS scales and constants chosen were pH on NBS scale; K_1_ and K_2_ from Mehrbach et al. (1973) (GEOSECS constants) for salinity ≥1 psu, and K_1_ and K_2_ from Millero (1979) for salinity <1 psu; K_SO4_ from Dickson et al. (1990); K_f_ from Perez and Fraga (1987); total boron from Lee et al. (2010). The same approach was used to calculate the range of dissolved CO_2_ concentrations under air-equilibrium conditions (assuming 400 ppm CO_2_ in air) across the different environmental temperatures and salinities.

Since most aquatic organisms might possess CCMs due to either permanent or temporal CO_2_ limitation for photosynthesis, a key question is whether these mechanisms elevate intracellular CO_2_ enough to almost reach the saturation of photosynthesis at ambient CO_2_ conditions. To address this, a literature compilation of the apparent photosynthetic semi-saturation constant for CO_2_ (K_m CO2_) measured under environmentally relevant conditions (temperature, pH, and salinity usually experimented by the organism) was performed in 154 species spanning the main taxonomic groups of oxygenic aquatic phototrophs from freshwater to marine environments (Cyanobacteria, Chlorophyta, Charophyta, Spermatophyta, Rhodophyta, Ochrophyta, Cryptophyta, Haptophyta, Euglenozoa, and Dinoflagellata). Figure 2 shows that, when all phylogenetic groups were considered together, species containing encapsulated Rubisco within a microcompartment exhibited significantly lower K_m CO2_ values (indicating higher apparent photosynthetic affinity for CO_2_ under air-equilibrium conditions), and no statistical difference was observed between freshwater and marine organisms, as previously proposed (Whitehead et al. 2014; Clement et al. 2017). When compared with dissolved CO_2_ concentrations under air-equilibrium conditions (calculated for the specific salinity and temperature of each measurement) and assuming that photosynthetic saturation requires 4 times the K_m CO2_ (Raven et al. 2017), the compiled dataset indicates that all cyanobacteria, nearly all diatoms and haptophytes, and approximately one-half of the analyzed dinoflagellates, chlorophytes, and rhodophytes achieve CO_2_-saturated photosynthesis under present atmospheric conditions (see Fig. 2 and Supplementary Dataset S2).

Saturated carbon fixation rates can be a result of 2 types of adaptation processes: increased CO_2_ availability at the sites of carboxylation, mainly by the appearance of CCMs, or intrinsic improvements in Rubisco carboxylation kinetics, by means of enhanced affinity and specificity for CO_2_. Almost all aquatic species able to reach CO_2_-saturated photosynthetic rates possess Rubisco-containing microcompartments (Fig. 2), consistent with reduced CO_2_ leakage out of the cell relative to organisms in which Rubisco is distributed throughout the chloroplastic stroma (Beer and Beardall 2025). By contrast, most of the species compiled belonging to the other phylogenetic groups, such as phaeophytes, chrysophytes, charophytes, euglenozoa, and submersed spermatophytes, cannot reach saturated photosynthetic rates at ambient CO_2_ conditions.

Nevertheless, there are many aquatic species that do not reach saturated photosynthetic rates but might be able to concentrate CO_2_ around Rubisco over ambient CO_2_ levels. To analyze this, CCM effectiveness was calculated as the ratio between the in vitro Rubisco semi-saturation constant for CO_2_ under air conditions ($K_{c}^{21\text{\%}\;O_{2}}$) and the in vivo apparent photosynthetic semi-saturation constant for CO_2_ (K_m CO2_), which indicates the fold changes that the CO_2_ concentration is increased or reduced near Rubisco active sites over the environmental CO_2_ level during steady-state photosynthesis (Raven et al. 2017). $K_{c}^{21\text{\%}\;O_{2}}$ was calculated using linear regression from K_c_ and K_o_ values at 25 °C, when not reported by literature, and normalized to the temperature at which K_m CO2_ was measured (see Supplementary Dataset S2), before calculating the CCM effectiveness $(K_{c}^{21\text{\%}\;O_{2}}$ /K_m CO2_ ratio).

**Figure 3:**
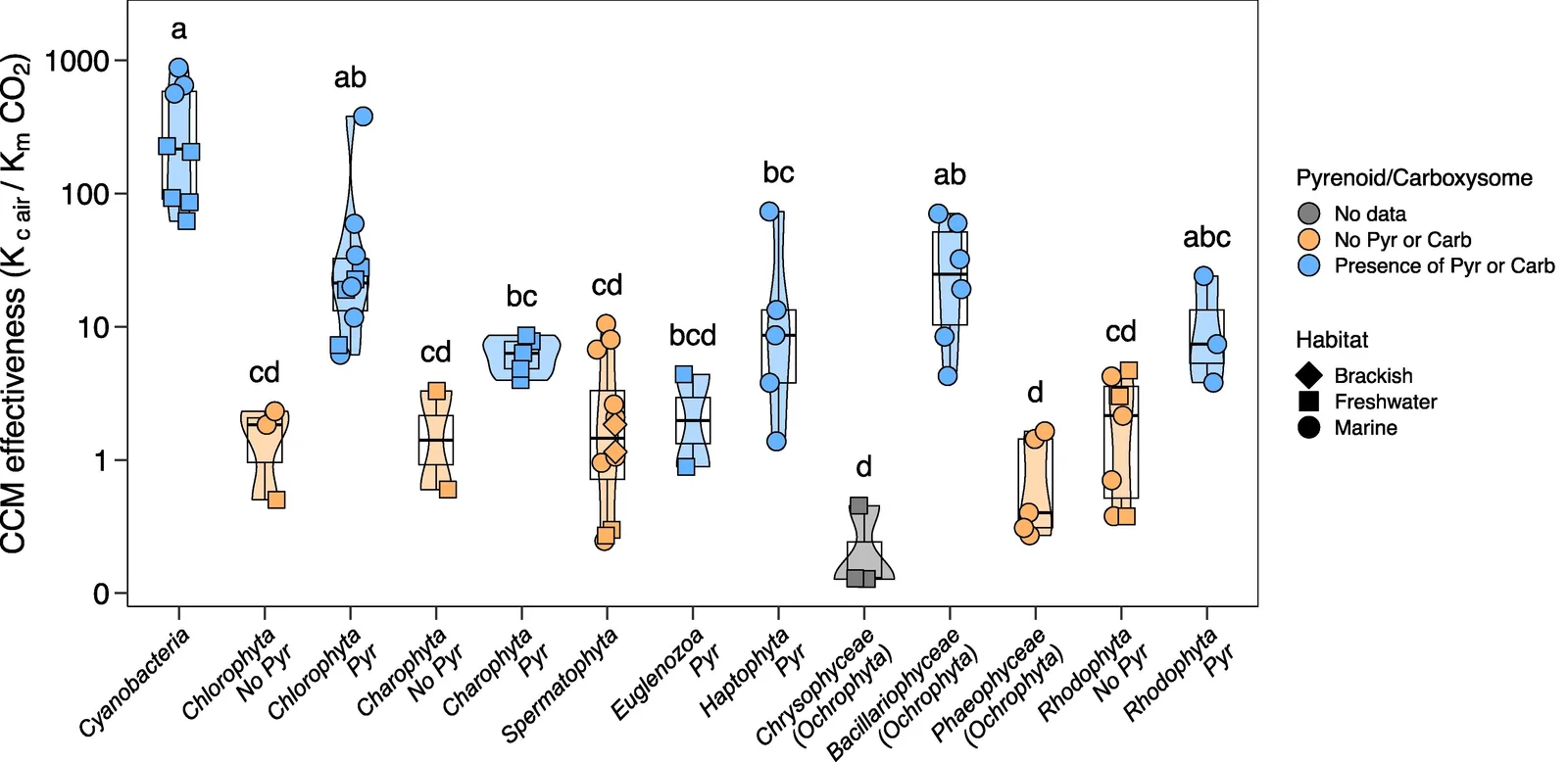
CCM effectiveness across aquatic oxygenic photosynthetic lineages. Violin plots show the distribution of CCM effectiveness (in log scale), calculated as the ratio between the Rubisco semi-saturation constant for CO_2_ under 21% O_2_ (K_c air_) and the apparent photosynthetic semi-saturation constant for CO_2_ (K_m CO2_; Fig. 2), ie K_c air_/K_m CO2_. K_c air_ at 25 °C (Fig. S1) was recalculated at the temperature used in the K_m CO2_ determination by using the thermal equations described in Galmés et al. (2019). In the few cases where the K_c air_ value was not available, K_c_ was used instead for the calculation of the mentioned ratio. The K_c air_/K_m CO2_ ratio represents the fold increase in CO_2_ availability at Rubisco active sites relative to the external CO_2_ concentration during steady-state photosynthesis. Embedded boxplots indicate the median (central line), interquartile range (25th–75th percentiles; box), and whiskers represent the data range excluding outliers. Points represent individual species and are colored according to the presence (blue) or absence (orange) of a Rubisco-containing microcompartment (pyrenoid in eukaryotic algae or carboxysome in Cyanobacteria); gray points indicate species for which the information about the presence/absence of pyrenoids was not available. The shape of the points represents the habitat of the species: marine (circle), freshwater (square), and brackish (diamond). Different letters indicate significant differences among groups (Kruskal–Wallis test followed by pairwise comparisons with Bonferroni correction; *P* < 0.05).

As expected, the highest CCM effectiveness occurred in those species being able to reach almost saturated photosynthesis at ambient CO_2_ conditions, for instance, Cyanobacteria, diatoms, haptophytes, and some rhodophytes and chlorophytes expressing pyrenoids (Fig. 3). Notably, none of the compiled species could achieve CO_2_-saturated photosynthesis solely through Rubisco kinetics without CCM operation. Only those organisms with relatively high Rubisco affinity for CO_2_ inhabiting cold waters (under 4 °C) could, theoretically, reach saturated photosynthesis at air-equilibrated CO_2_ levels only with passive CO_2_ diffusion, since K_c_ and S_c/o_ decrease at low temperatures, whereas dissolved CO_2_ and the ratio CO_2_/O_2_ increase (Iñiguez et al. 2019). Despite this, even polar photosynthetic organisms have been shown to use bicarbonate for photosynthesis, likely due to the lower diffusion of CO_2_ and lower uncatalyzed rate of CO_2_ supply from bicarbonate in cold waters (Iñiguez et al. 2019). Other compiled aquatic species are able to effectively concentrate CO_2_ around Rubisco by 3- to 8-fold the environmental CO_2_ levels, although not reaching CO_2_-saturated photosynthetic rates, such as most charophytes, many seagrasses, and some chlorophytes and rhodophytes, including species with and without pyrenoids (Fig. 3). Examples of them are the thermoacidophilic rhodophyte *C. merolae*, the seaweed *Anadyomene stellata* and *Ellisolandia elongata*, and the seagrasses *P. oceanica*, *Cymodocea nodosa*, and *Zostera noltii.* These observations indicate that pyrenoids seem not to be a prerequisite for effectively concentrate CO_2_ around Rubisco, albeit the present compilation shows that CCMs based on Rubisco packaged in microcompartments (either carboxysomes or pyrenoids) are, on average, significantly more effective than other types of aquatic CCMs present in oxygenic photoautotrophs (Fig. S2).

For peridinin-containing dinoflagellates, CCM effectiveness cannot be directly determined because kinetic parameters remain largely unknown for their form II Rubiscos, aside from a specificity value measured in *Amphidinium carterae* at 10 °C, due to enzyme instability after extraction (Whitney et al. 1998). However, assuming K_c_^air^ values similar to those reported for other form II Rubiscos, such as that of the proteobacterium *Rhodospirillum rubrum*, yields an estimated mean CCM effectiveness of ∼30. This estimate suggests the presence of highly effective CCMs, consistent with direct in vivo intracellular DIC accumulation measurements reported for dinoflagellates (Raven et al. 2020).

Despite the permanent CO_2_ limitation in marine environments compared with the greater fluctuation of dissolved CO_2_ in freshwater environments, no significant differences were observed in the potential CCM effectiveness between freshwater and marine organisms when all phylogenetic groups were considered together (Fig. S2). Nevertheless, few taxa with compiled organisms from both marine and freshwater environments show a trend for higher CCM effectiveness in marine relative to freshwater species. This is the case for submersed angiosperms, where only marine species, and no freshwater species, are able to effectively concentrate CO_2_ around Rubisco over environmental CO_2_ levels (Capó-Bauçà et al. 2022).

Bicarbonate photosynthetic utilization, either directly or indirectly, has been widely demonstrated across aquatic organisms by proxies including pH drift assays, biomass carbon isotopic discrimination, and carbon isotope disequilibrium via membrane-inlet mass spectrometry (Madsen and Sand-Jensen 1991; Maberly et al. 1992; Badger et al. 1994; Raven et al. 2002; Rost et al. 2003), whereas only a reduced number of species appear to rely primarily on passive CO_2_ diffusion for photosynthesis. These include chrysophytes, likely reflecting their preference for CO_2_-enriched flowing waters, like streams, that reduce selective pressure for CCM evolution (Maberly et al. 2009). Other examples are the freshwater rhodophyte *Lemanea mamillosa*, also inhabiting streams (MacFarlane and Raven 1985); the chlorophyte photobiont *Coccomyxa* (Palmqvist et al. 1994), where host respiration and restricted gas diffusion may enhance CO_2_ availability; and species from low light/shaded environments, such as lower-sublittoral seaweeds, in which photosynthesis is primarily constrained by irradiance rather than by Rubisco carboxylation (Johnston et al. 1992; Kübler and Raven 1994; Iñiguez et al. 2019).

However, the capacity to utilize HCO_3_^−^ for photosynthesis does not necessarily imply that CCMs are able to concentrate CO_2_ around Rubisco over environmental levels. In fact, CCMs can help alleviate cellular resistance to CO_2_ diffusion from the external medium to Rubisco active sites. In those cases, CCM effectiveness would have a value lower than 2 to 3 (Raven et al. 2017), considering the resistance to CO_2_ diffusion from the outside of the cell to Rubisco that has been previously estimated for C_3_ terrestrial embryophytes (Raven and Beardall 2016). This is the case for all the compiled phaeophytes, some red and green macroalgae and many submerged angiosperms (Maberly and Gontero 2017; Iñiguez et al. 2019; Capó-Bauçà et al. 2022, 2023a) (Fig. 3).

The interpretation of the apparent photosynthetic CO_2_ affinities and CCM effectiveness comes with some limitations. Dissolved CO_2_ in freshwater and productive coastal ecosystems is frequently not in equilibrium with the atmosphere, meaning that air-equilibrated CO_2_ may not reflect in situ CO_2_ availability. Moreover, CCMs based on localized periplasmic acidification are buffer sensitive, and the use of external pH buffers in photosynthesis-DIC curves may underestimate apparent CO_2_ affinity in those species possessing this mechanism (Price and Badger 1985; Hellblom et al. 2001; see Supplementary Dataset S2). Finally, K_m CO2_ values, derived from the fraction of the DIC pool at a given pH, temperature, and salinity, can be largely determined by HCO_3_^−^ uptake in species capable of HCO_3_^−^ use. Even so, comparisons between the apparent photosynthetic affinity for CO_2_ and in vitro Rubisco affinity for CO_2_ generally indicate that ambient CO_2_ is insufficient to sustain photosynthesis without intracellular CO_2_ enrichment, supporting the use of K_c_^air^/K_m CO2_ as a good indicator of CCM effectiveness.

### Coevolution of CCMs effectiveness and Rubisco kinetic traits

A previous meta-analysis pointed to a general relaxation of Rubisco CO_2_ affinity and specificity when coevolved with CCMs, with the evolutionary pressure shifted toward increasing k_cat_^c^ (Iñiguez et al. 2020). In the present study, Fig. 3 has revealed that there is an ample range of variation in CCM effectiveness among aquatic organisms, even within the same phylogenetic group (note that the Y axis is in logarithmic scale), highlighting the need for a deeper reevaluation of the coadaptation between aquatic CCMs and Rubisco kinetics.

Previous data from phylogenetically close aquatic species with contrasting CCM effectiveness suggest that the different Rubisco kinetic parameters might not have the same evolutive time scales. For instance, S_c/o_ seems to be quite constant within the same phylogenetic group (Flamholz et al. 2019), whereas Rubisco affinity for CO_2_ and carboxylation efficiency (and presumably Rubisco affinity for O_2_ and oxygenation efficiency) show substantial variation associated with contrasting environmental conditions and CCM performance (Capó-Bauçà et al. 2022, 2023a, 2023b).

**Figure 4:**
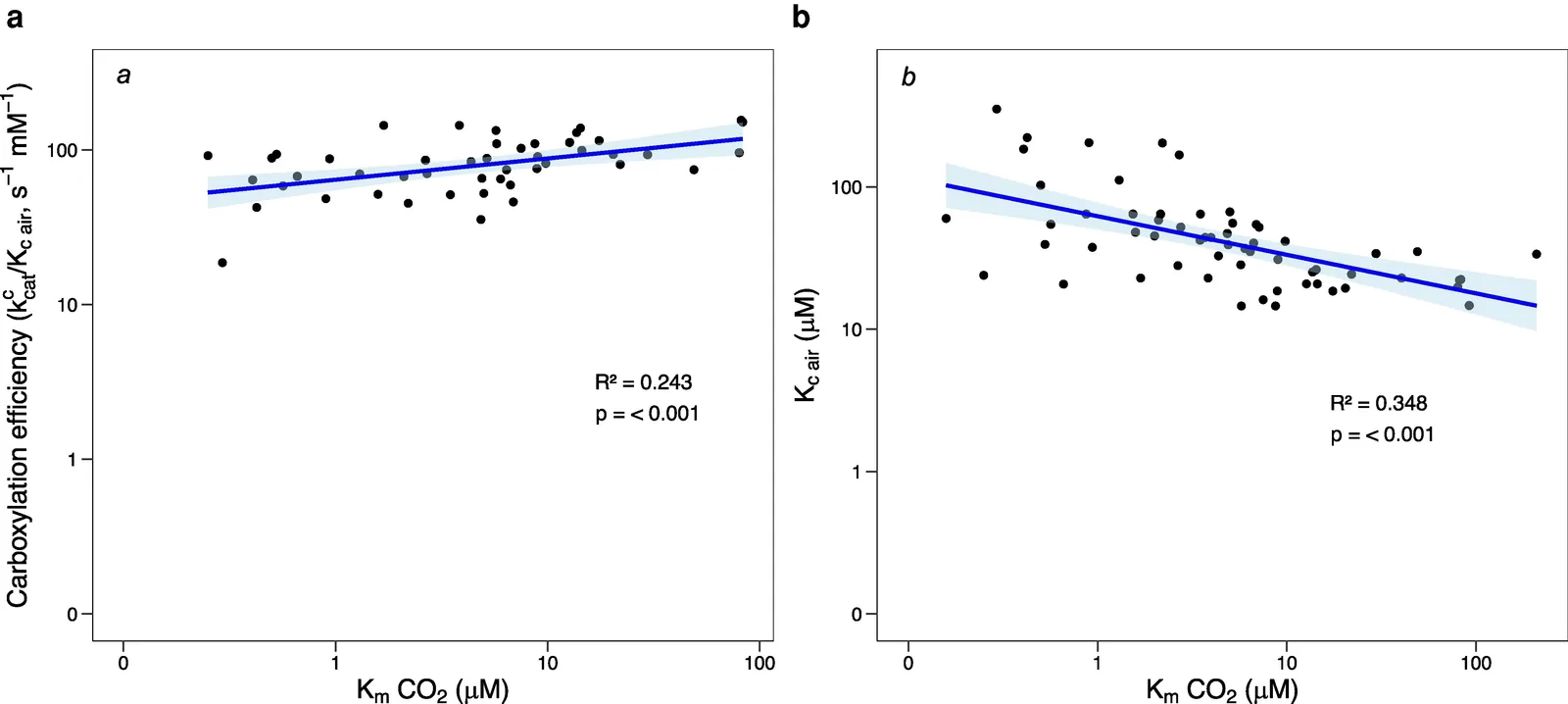
a) Relationship between the apparent photosynthetic semi-saturation constant for CO_2_ (K_m CO2_, µM) and Rubisco carboxylation efficiency under 21% oxygen (k_cat_^c^/K_c air_, s^−1^ mM^−1^) (n = 47). b) Relationship between the apparent photosynthetic semi-saturation constant for CO_2_ (K_m CO2_, µM) and the Rubisco semi-saturation constant for CO_2_ under 21% O_2_ (K_c air_, µM) (n = 60). Both axes are log_10_-transformed in both panels. The blue lines represent the linear regression with 95% confidence interval (shaded area). *R^2^* is the Pearson correlation coefficient and *p* the *P*-value of the Pearson correlation test.

In fact, an inverse relationship between Rubisco carboxylation efficiency under air conditions and the apparent photosynthetic CO_2_ affinity (and therefore, CCM effectiveness) has been previously proposed for aquatic macrophytes (Capó-Bauçà et al. 2024). Since Rubisco carboxylation efficiency and the apparent photosynthetic semi-saturation constant for CO_2_ (K_m CO2_; the inverse of the photosynthetic CO_2_ affinity) varies by orders of magnitude among the diverse aquatic organisms compiled in the present study, both variables were log-transformed to linearize the relationship expected from multiplicative physiological interactions and to meet the assumptions of linear regression (Fig. 4a). The significant positive slope in the log-log relationship shown in Fig. 4a agrees well with a direct potential association, with lower K_m CO2_ (ie higher apparent photosynthetic CO_2_ affinity) observed in organisms exhibiting lower Rubisco carboxylation efficiency (R^2^ = 0.24; n = 47; *P* < 0.001). In addition, the significant log-log relationship between K_c_^air^ and K_m CO2_ shown in Fig. 4b reinforces the hypothesis of a negative trade-off between photosynthetic and in vitro Rubisco affinities for CO_2_ in aquatic organisms (R^2^ = 0.35; n = 60; *P* < 0.001). Regarding S_c/o_, its relationship with the apparent photosynthetic affinity for CO_2_ was not significant (data not shown), supporting the hypothesis of a longer evolutive time scale for this kinetic parameter in aquatic groups, that seems to be more phylogenetically constrained than Rubisco CO_2_ affinity and carboxylation efficiency. This evolutionary decoupling might explain the absence of canonical trade-offs between Rubisco catalytic traits (eg k_cat_^c^ vs K_c_ or S_c/o_ vs K_c_) reported in several aquatic phylogenetic groups (Young et al. 2016; Iñiguez et al. 2019, 2020; Goudet et al. 2020; Capó-Bauçà et al. 2022, 2023a, 2023b).

In terrestrial ecosystems, the relaxation in the Rubisco affinity for CO_2_ observed in plants expressing biochemical CCMs (C_4_ or CAM metabolisms), relative to their C_3_ counterparts, is accompanied by an enhancement in k_cat_^c^ that provoke a maintenance in carboxylation efficiency, together with a relaxation in S_c/o_ (Sharwood et al. 2016), thereby increasing Rubisco oxygenation efficiency. Since most photosynthetic O_2_ production in the main subtype of C_4_ plants, NADP-ME, is spatially separated from Rubisco activity, enhanced oxygenation efficiency does not necessarily compromise net carbon fixation. Conversely, in aquatic organisms with effective CCMs, the reduction in the Rubisco affinity for O_2_ and oxygenation efficiency may represent an adaptive response to elevated intracellular O_2_ levels arising from restricted gas diffusion during submerged photosynthesis. Oxygen availability around Rubisco may therefore constitute an important driver shaping Rubisco kinetic traits in aquatic environments, highlighting a fundamental divergence between terrestrial and aquatic trajectories of Rubisco-CCM coevolution.

### CCM operation under future global change scenarios

The main changes in the marine environment driven by the anthropogenic causes potentially affecting CCMs are the increase in the dissolved concentration of CO_2_ and bicarbonate, the increase in temperature, and the decrease in pH (ocean acidification). The increase in atmospheric levels of CO_2_ and the concomitant increase in temperature act as antagonist in terms of the resulting dissolved CO_2_ levels in seawater. The solubility of CO_2_ decreases with temperature, and, for instance, an increase from 10 °C to 15 °C leads to a 15% decrease in the dissolved CO_2_ concentration in seawater (Table 1), while the increase in atmospheric CO_2_ from pre-industrial levels (280 ppm) to current levels (420 ppm) has caused an increase of 50% (when no temperature rise is considered). Thus, the incremental effect by atmospheric CO_2_ outpaces the decrease expected by the rise in temperature. In general, CCMs are regulated by the external CO_2_ levels so that an increase in CO_2_ typically decreases the activity of CCMs in both microalga (Giordano et al. 2005) and macrophytes (van der Loos et al. 2018). Since CCMs are energetically demanding, a lower CCM activity would free energy that can be invested into growth (Gordillo et al. 2003; van der Loos et al. 2018). However, this inactivation does not always take place. In Arctic seaweeds, most of the tested species did not downregulate external carbonic anhydrase activity in response to increased external CO_2_ (Gordillo et al. 2006, 2016; Iñiguez et al. 2016). Since dissolved CO_2_ is already high in air-equilibrated polar waters due to its higher solubility at low temperature, it was suspected that the lack of inhibition by CO_2_ might be related to the role of CCMs in mitigating photoinhibition, particularly under constant solar radiation during the polar summer. This seems to be the case at least for *Desmarestia anceps* (Iñiguez et al. 2017). The operation of CCMs at high CO_2_ may not be restricted to polar seaweeds, as it has also been observed in polar diatoms (Cassar et al. 2004; Tortell et al. 2008).

**Table 1:** Air equilibrium concentrations of dissolved CO_2_/HCO_3_^−^ (µmol/kg SW) in seawater at different temperatures and different levels of atmospheric CO_2_ concentrations (ppm), assuming a total alkalinity of 2300 µequivalents L^−1^, and a salinity of 33 psu.

	2 °C	7 °C	10 °C	15 °C	20 °C
280 ppm	16.4/1982	13.7/1921	12.4/1882	10.6/1814	9.1/1742
420 ppm	24.6/2068	20.6/2020	18.6/1989	15.9/1932	13.7/1871
900 ppm	52.8/2180	44.1/2151	39.8/2133	34/2098	29.4/2058

The growth success of aquatic autotrophs to increased CO_2_ cannot be inferred only from their inorganic carbon uptake mechanisms. High CO_2_ levels can modify the gene expression of molecular mechanisms involved in light harvesting, nitrate assimilation, and ATP synthesis (Li et al. 2015). In the green seaweed *Ulva sp.*, high CO_2_ stimulated the nitrate reductase activity (Gordillo et al. 2001). These changes can potentially modify the energetics of carbon assimilation and influence the operation of CCMs in various ways (Burlacot and Peltier 2023), which in turn could be responsible for the wide array of species-specific responses observed both in micro and macroalgae as well as seagrasses (Reinfelder 2011; van der Loos et al. 2018). For instance, growth performance can be benefited by a decrease in respiration rates at high CO_2_, thereby favoring a net positive C balance without affecting photosynthesis (Gordillo et al. 2003; Iñiguez et al. 2016).

Changes in CCM activity with rising temperature display a wide variety of responses that might be a consequence of indirect effects on other metabolic processes. Greenhouse warming of the ocean surface results in enhanced stratiﬁcation and shoaling of upper mixed layers, exposing photosynthetic organisms dwelling there to increased light and UV radiation as well as to a decreased nutrient supply, so that changes in CCM activity can be attributed to lower nutrient availability or increased light irradiance (Sobrino et al. 2008; Gao et al. 2019). It is still to be determined if CCM activity can play a role under these circumstances by mitigating photoinhibition.

Light limitations for photosynthesis occurring in benthic organisms living at considerable depths make the energy investment required to operate a CCM disadvantageous. Most of the marine species lacking an active CCM belong to those environments (van der Loos et al. 2018; Fernández et al. 2024). Even though increased CO_2_ could potentially benefit non-CCM species over CCM ones, this will rarely be the case for light-limiting environments. Changes in the light environment may well happen due to global warming. The ongoing temperature rise in the coastal areas of both polar regions, usually dominated by seaweed forests in rocky areas, has shortened the darkness period due to the disappearance of an ice cover, increasing the annual light dose and thus causing a dramatic shift in the community composition (Düsedau et al. 2024). Regarding freshwater environments, annual average concentrations of CO_2_ are oversaturated in most lakes and rivers (Cole et al. 1994; Raymond et al. 2013) because of microbial activity and photodegradation of organic carbon produced on land (Sobek et al. 2007) and within the water body (Maberly et al. 2013). The direct effect of an increase in soluble CO_2_ levels in these systems is not likely to be great. However, given the huge variability in alkalinity and pH, particular catchment conditions may trigger changes in CCM operation that can modify the adequacy and competitive factor of some species over others, leading to potential changes at the community level.

Overall, higher levels of dissolved CO_2_ are expected to favor non-CCM but also some CCM species if the freed energy can be invested into biomass. The final result in growth performance of a given species will also depend on the effects of ambient CO_2_ levels on metabolic processes other than CCMs and the co-occurring changes in other environmental factors such as light and nutrient availability that indirectly modify CCM activity.

## Concluding remarks

The synthesis presented here highlights that, despite the diversity of Rubisco forms and CCM structures/components across aquatic photoautotrophs, there is a clear functional convergence toward similarly high apparent photosynthetic CO_2_ affinity among a wide range of taxa in both marine and freshwater environments. Their ability to operate CO_2_-saturated photosynthesis under air-equilibrated dissolved CO_2_ levels is, in most cases, due to highly effective CCMs based on Rubisco-containing microcompartments in the cytosol (cyanobacterial carboxysomes) or in the chloroplastic stroma (algal pyrenoids). This indicates that aquatic environments have promoted the evolution toward increased CO_2_ availability at the sites of carboxylation rather than improvements in Rubisco carboxylation kinetics, contrarily to what has been shown for C_3_ terrestrial plants (Bouvier et al. 2024). Finally, the inverse relationship between Rubisco carboxylation efficiency and the apparent photosynthetic CO_2_ affinity (and therefore, CCM effectiveness) point to a divergent trajectory of Rubisco–CCM coevolution in aquatic systems relative to terrestrial plants. Together, these findings emphasize that the effectiveness of CCMs, rather than their structural diversity or their ability to use bicarbonate, is a key determinant on the ecophysiology of aquatic photosynthesis.

Still, many gaps of knowledge persist regarding the resistance to CO_2_ influx and O_2_ efflux across cellular membranes in microorganisms (Cyanobacteria, green and non-green microalgae) and submersed macrophytes that differentially constrain aquatic CO_2_ assimilation. In addition, CCM effectiveness remains largely unexplored in entire phylogenetic groups such as cryptophytes, dinoflagellates, xantophytes, raphydophytes, and bolidophytes, limiting the possibility to fully resolve the complete landscape of aquatic CCM functioning.

## Author contributions

Data were compiled by C.I., P.C., and P.A.N. Additional correction and standardization of Rubisco kinetic parameters were done by J.G. and C.I. Figures were generated by S.C.B. The manuscript was written by C.I. with contributions from all coauthors.

## Supplementary Material

kiag507_Supplementary_Data

## Data Availability

All data cited in this review are available in the referenced publications. No new data were generated.

## References

[kiag507-B1] Aguiló-Nicolau P et al 2023. Singular adaptations in the carbon assimilation mechanism of the polyextremophile cyanobacterium *Chroococcidiopsis thermalis*. Photosynth Res. 156:231–245. 10.1007/s11120-023-01008-y. 36941458 PMC10154277

[kiag507-B2] Aguiló-Nicolau P et al Diversity in Rubisco Kinetics and CO2-concentrating mechanisms among Cyanobacterial lineages [preprint]. bioRxiv 2025. 10.64898/2025.12.30.696979. PMC13281935 42060838

[kiag507-B3] Badger MR et al 1998. The diversity and coevolution of Rubisco, plastids, pyrenoids, and chloroplast-based CO_2_-concentrating mechanisms in algae. Can J Bot. 76:1052–1071. 10.1139/b98-074

[kiag507-B4] Badger MR, Palmqvist K, Yu J-W. 1994. Measurement of CO_2_ and HCO_3_^−^ fluxes in cyanobacteria and microalgae during steady-state photosynthesis. Physiol Plant. 90:529–536. 10.1111/j.1399-3054.1994.tb08811.x.

[kiag507-B5] Barrett J, Girr P, Mackinder LCM. 2021. Pyrenoids: CO_2_-fixing phase separated liquid organelles. Biochim Biophys Acta Mol Cell Res. 1868:118949. 10.1016/j.bbamcr.2021.118949. 33421532

[kiag507-B6] Barrett J et al 2024. A promiscuous mechanism to phase separate eukaryotic carbon fixation in the green lineage. Nat Plants. 10:1801–1813. 10.1038/s41477-024-01812-x. 39384944 PMC11570498

[kiag507-B7] Beer S, Beardall J. 2025. Inorganic carbon acquisition and photosynthetic metabolism in marine photoautotrophs: a summary. Plants. 14:904. 10.3390/plants14060904. 40265828 PMC11944913

[kiag507-B8] Bouvier JW et al 2021. Rubisco adaptation is more limited by phylogenetic constraint than by catalytic trade-off. Mol Biol Evol. 38:2880–2896. 10.1093/molbev/msab079. 33739416 PMC8233502

[kiag507-B9] Bouvier JW, Emms DM, Kelly S. 2024. Rubisco is evolving for improved catalytic efficiency and CO_2_ assimilation in plants. Proc Natl Acad Sci U S A. 121:e2321050121. 10.1073/pnas.2321050121. 38442173 PMC10945770

[kiag507-B10] Burlacot A, Peltier G. 2023. Energy crosstalk between photosynthesis and the algal CO_2_-concentrating mechanisms. Trends Plant Sci. 28:795–807. 10.1016/j.tplants.2023.03.018. 37087359

[kiag507-B11] Cabello-Yeves PJ et al 2022. α-cyanobacteria possessing form IA RuBisCO globally dominate aquatic habitats. ISME J. 16:2421–2432. 10.1038/s41396-022-01282-z. 35851323 PMC9477826

[kiag507-B12] Capó-Bauçà S, Galmés J, Aguiló-Nicolau P, Ramis-Pozuelo S. 2023a. Iñiguez C. Carbon assimilation in upper subtidal macroalgae is determined by an inverse correlation between rubisco carboxylation efficiency and CO_2_ concentrating mechanism effectiveness. New Phytol. 237:2027–2038. 10.1111/nph.18623. 36385703

[kiag507-B13] Capó-Bauçà S, Iñiguez C, Aguiló-Nicolau P, Galmés J. 2022. Correlative adaptation between rubisco and CO_2_-concentrating mechanisms in seagrasses. Nat Plants. 8:706–716. 10.1038/s41477-022-01171-5. 35729266

[kiag507-B14] Capó-Bauçà S, Iñiguez C, Galmés J. 2024. The diversity and coevolution of Rubisco and CO_2_ concentrating mechanisms in marine macrophytes. New Phytol. 241:2353–2365. 10.1111/nph.19528. 38197185

[kiag507-B15] Capó-Bauçà S et al 2023b. The trajectory in catalytic evolution of Rubisco in *Posidonia* seagrass species differs from terrestrial plants. Plant Physiol. 191:946–956. 10.1093/plphys/kiac492. 36315095 PMC9922400

[kiag507-B16] Catherall E et al 2025. From algae to plants: understanding pyrenoid-based CO_2_-concentrating mechanisms. Trends Biochem Sci. 50:33–45. 10.1016/j.tibs.2024.10.010. 39592300

[kiag507-B17] Casati P, Lara MV, Andreo CS. 2000. Induction of a C_4_-like mechanism of CO_2_ fixation in *Egeria densa*, a submersed aquatic species. Plant Physiol. 123:1611–1622. 10.1104/pp.123.4.1611. 10938377 PMC59118

[kiag507-B18] Cassar N, Laws EA, Bidigare RR, Popp BN. 2004. Bicarbonate uptake by Southern Ocean phytoplankton. Global Biogeochem Cycles. 18:GB2003. 10.1029/2003GB002116.

[kiag507-B19] Clement R, Jensen E, Prioretti L, Maberly SC, Gontero B. 2017. Diversity of CO_2_-concentrating mechanisms and responses to CO_2_ concentration in marine and freshwater diatoms. J Exp Bot. 68:3925–3935. 10.1093/jxb/erx035. 28369472

[kiag507-B20] Cole JJ, Caraco NF, Kling GW, Kratz TK. 1994. Carbon dioxide supersaturation in the surface waters of lakes. Science. 265:1568–1570. 10.1126/science.265.5178.1568. 17801536

[kiag507-B21] Cummins PL, Kannappan B, Gready JE. 2018. Directions for optimization of photosynthetic carbon fixation: ruBisCO's efficiency may not be so constrained after all. Front Plant Sci. 9:183. 10.3389/fpls.2018.00183. 29545812 PMC5838012

[kiag507-B22] Dickson AG, Wesolowski DJ, Palmer DA, Mesmer RE. 1990. Dissociation constant of bisulfate ion in aqueous sodium chloride solutions to 250℃. J Phys Chem. 94:7978–7985. 10.1021/j100383a042.

[kiag507-B23] Düsedau L et al 2024. Kelp forest community structure and demography in Kongsfjorden (Svalbard) across 25 years of Arctic warming. Ecol Evol. 14:e11606. 10.1002/ece3.11606. 38919650 PMC11199086

[kiag507-B24] Fernández PA et al 2024. Diverse inorganic carbon uptake strategies in Antarctic seaweeds: revealing species-specific responses and implications for ocean acidification. Sci Total Environ. 945:174006. 10.1016/j.scitotenv.2024.174006. 38889822

[kiag507-B25] Flamholz AI et al 2019. Revisiting trade-offs between Rubisco kinetic parameters. Biochemistry. 58:3365–3376. 10.1021/acs.biochem.9b00237. 31259528 PMC6686151

[kiag507-B26] Flynn KJ et al 2012. Changes in pH at the exterior surface of plankton with ocean acidification. Nat Clim Change. 2:510–513. 10.1038/nclimate1489.

[kiag507-B27] Galmés J et al 2014. Rubisco catalytic properties optimized for present and future climatic conditions. Plant Sci. 226:61–70. 10.1016/j.plantsci.2014.01.008. 25113451

[kiag507-B28] Galmés J, Capó-Bauçà S, Niinemets Ü. Iñiguez C. 2019. Potential improvement of photosynthetic CO_2_ assimilation in crops by exploiting the natural variation in the temperature response of Rubisco catalytic traits. Curr Opin Plant Biol. 49:60–67. 10.1016/j.pbi.2019.05.002. 31234057

[kiag507-B29] Galmés J, Hermida-Carrera C, Laanisto L, Niinemets Ü. 2016. A compendium of temperature responses of Rubisco kinetic traits: variability among and within photosynthetic groups and impacts on photosynthesis modeling. J Exp Bot. 67:5067–5091. 10.1093/jxb/erw267. 27406782 PMC5014154

[kiag507-B30] Gao K et al 2019. Effects of ocean acidification on marine photosynthetic organisms under the concurrent influences of warming, UV radiation, and deoxygenation. Front Mar Sci. 6:322. 10.3389/fmars.2019.00322.

[kiag507-B31] Giordano M, Beardall J, Raven JA. 2005. CO_2_ concentrating mechanisms in algae: mechanisms, environmental modulation, and evolution. Annu Rev Plant Biol. 56:99–131. 10.1146/annurev.arplant.56.032604.144052. 15862091

[kiag507-B32] Gordillo FJL, Aguilera J, Jiménez C. 2006. The response of nutrient assimilation and biochemical composition of Arctic seaweeds to a nutrient input in summer. J Exp Bot. 57:2661–2671. 10.1093/jxb/erl029. 16829547

[kiag507-B33] Gordillo FJL, Carmona R, Viñegla B, Wiencke C, Jiménez C. 2016. Effects of simultaneous increase in temperature and ocean acidification on biochemical composition and photosynthetic performance of common macroalgae from Kongsfjorden (Svalbard). Polar Biol. 39:1993–2007. 10.1007/s00300-016-1897-y.

[kiag507-B34] Gordillo FJL, Figueroa FL, Niell FX. 2003. Photon- and carbon-use efficiency in *Ulva rigida* at different CO_2_ and N levels. Planta. 218:315–322. 10.1007/s00425-003-1087-3. 12937985

[kiag507-B35] Gordillo FJL, Niell F, Figueroa F. 2001. Non-photosynthetic enhancement of growth by high CO_2_ level in the nitrophilic seaweed *Ulva rigida* C. Agardh (Chlorophyta). Planta. 213:64–70. 10.1007/s004250000468. 11523657

[kiag507-B36] Goudet MMM et al 2020. Rubisco and carbon-concentrating mechanism co-evolution across chlorophyte and streptophyte green algae. New Phytol. 227:810–823. 10.1111/nph.16577. 32249430

[kiag507-B37] Heise CM et al 2025. Evidence for a CO_2_-concentrating mechanism in the model streptophyte green alga *Chara braunii*. New Phytol. 247:1218–1233. 10.1111/nph.70283. 40515453 PMC12222935

[kiag507-B38] Hellblom F, Beer S, Björk M, Axelsson L. 2001. A buffer sensitive inorganic carbon utilisation system in *Zostera marina*. Aquat Bot. 69:55–62. 10.1016/S0304-3770(00)00132-7.

[kiag507-B39] Heureux AMC et al 2017. The role of Rubisco kinetics and pyrenoid morphology in shaping the CCM of haptophyte microalgae. J Exp Bot. 68:3959–3969. 10.1093/jxb/erx179. 28582571 PMC5853415

[kiag507-B40] Hopkinson BM, Dupont CL, Matsuda Y. 2016. The physiology and genetics of CO_2_ concentrating mechanisms in model diatoms. Curr Opin Plant Biol. 31:51–57. 10.1016/j.pbi.2016.03.013. 27055267

[kiag507-B41] Iñiguez C et al 2016. Increased temperature, rather than elevated CO_2_, modulates the carbon assimilation of the Arctic kelps *Saccharina latissima* and *Laminaria solidungula*. Mar Biol. 163:248. 10.1007/s00227-016-3024-6.

[kiag507-B42] Iñiguez C et al 2020. Evolutionary trends in RuBisCO kinetics and their co-evolution with CO_2_ concentrating mechanisms. Plant J. 101:897–918. 10.1111/tpj.14643. 31820505

[kiag507-B43] Iñiguez C, Galmés J, Gordillo FJL. 2019. Rubisco carboxylation kinetics and inorganic carbon utilization in polar versus cold-temperate seaweeds. J Exp Bot. 70:1283–1297. 10.1093/jxb/ery443. 30576461 PMC6382342

[kiag507-B44] Iñiguez C, Heinrich S, Harms L, Gordillo FJL. 2017. Increased temperature and CO_2_ alleviate photoinhibition in *Desmarestia anceps*: from transcriptomics to carbon utilization. J Exp Bot. 68:3971–3984. 10.1093/jxb/erx164. 28575516 PMC5853390

[kiag507-B45] Iñiguez C, Niinemets Ü, Mark K, Galmés J. 2021. Analyzing the causes of method-to-method variability among Rubisco kinetic traits: from the first to the current measurements. J Exp Bot. 72:7846–7862. 10.1093/jxb/erab356. 34329386

[kiag507-B46] Jaffe AL et al 2024. Cyanobacteria from marine oxygen-deficient zones encode both form I and form II Rubiscos. Proc Natl Acad Sci U S A. 121:e2418345121. 10.1073/pnas.2418345121. 39585972 PMC11626144

[kiag507-B47] Jiang HS et al 2026. A novel single-cell NAD-ME C_4_ subtype integrated with CAM and bicarbonate use in an aquatic plant. New Phytol. 249:2386–2401. 10.1111/nph.70673. 41121679 PMC12873514

[kiag507-B48] Johnston AM, Maberly SC, Raven JA. 1992. The acquisition of inorganic carbon by four red macroalgae. Oecologia. 92:317–326. 10.1007/BF00317457. 28312597

[kiag507-B49] Kübler JE, Raven JA. 1994. Consequences of light limitation for carbon acquisition in three rhodophytes. Mar Ecol Prog Ser. 110:203–209. 10.3354/meps110203.

[kiag507-B50] Lee K et al 2010. The universal ratio of boron to chlorinity for the North Pacific and North Atlantic oceans. Geochim Cosmochim Acta. 74:1801–1811. 10.1016/j.gca.2009.12.027.

[kiag507-B51] Li Y et al 2015. Ocean acidification modulates expression of genes and physiological performance of a marine diatom. Biogeosci Discuss. 12:15809–15833. 10.5194/bgd-12-15809-2015 PMC5305191 28192486

[kiag507-B52] Long BM, Matsuda Y, Moroney JV. 2024. Algal chloroplast pyrenoids: evidence for convergent evolution. Proc Natl Acad Sci U S A. 121:e2402546121. 10.1073/pnas.2402546121. 38513078 PMC10998615

[kiag507-B53] Maberly SC, Ball LA, Raven JA, Sültemeyer D. 2009. Inorganic carbon acquisition by chrysophytes. J Phycol. 45:1052–1061. 10.1111/j.1529-8817.2009.00734.x. 27032350

[kiag507-B54] Maberly SC, Barker PA, Stott AW, De Ville MM. 2013. Catchment productivity controls CO_2_ emissions from lakes. Nat Clim Change. 3:391–394. 10.1038/nclimate1748.

[kiag507-B55] Maberly SC, Gontero B. 2017. Ecological imperatives for aquatic CO_2_-concentrating mechanisms. J Exp Bot. 68:3797–3814. 10.1093/jxb/erx201. 28645178

[kiag507-B56] Maberly SC, Raven JA, Johnston AM. 1992. Discrimination between ^12^C and ^13^C by marine plants. Oecologia. 91:481–492. 10.1007/BF00650320. 28313499

[kiag507-B57] MacFarlane JJ, Raven JA. 1985. External and internal CO_2_ transport in *Lemanea*: interactions with the kinetics of ribulose bisphosphate carboxylase. J Exp Bot. 36:610–622. 10.1093/jxb/36.4.610.

[kiag507-B58] Madsen TV, Sand-Jensen K. 1991. Photosynthetic carbon assimilation in aquatic macrophytes. Aquat Bot. 41:5–40. 10.1016/0304-3770(91)90037-6.

[kiag507-B59] Magnin NC, Cooley BA, Reiskind JB, Bowes G. 1997. Regulation and localization of key enzymes during the induction of Kranz-less, C_4_-type photosynthesis in *Hydrilla verticillata*. Plant Physiol. 115:1681–1689. 10.1104/pp.115.4.1681. 12223888 PMC158634

[kiag507-B60] Mass T, Genin A, Shavit U, Grinstein M, Tchernov D. 2010. Flow enhances photosynthesis in marine benthic autotrophs by increasing the efflux of oxygen from the organism to the water. Proc Natl Acad Sci U S A. 107:2527–2531. 10.1073/pnas.0912348107. 20133799 PMC2823876

[kiag507-B61] Mehrbach C, Culberson CH, Hawley JE, Pytkowicz RM. 1973. Measurement of the apparent dissociation constants of carbonic acid in seawater at atmospheric pressure. Limnol Oceanogr. 18:897–907. 10.4319/lo.1973.18.6.0897.

[kiag507-B62] Millero FJ . 1979. The thermodynamics of the carbonate system in seawater. Geochim Cosmochim Acta. 43:1651–1661. 10.1016/0016-7037(79)90184-4.

[kiag507-B63] Moromizato R et al 2024. Pyrenoid proteomics reveals independent evolution of the CO_2_-concentrating organelle in chlorarachniophytes. Proc Natl Acad Sci U S A. 121:e2318542121. 10.1073/pnas.2318542121. 38408230 PMC10927497

[kiag507-B64] Morse D, Salois P, Markovic P, Hastings JW. 1995. A nuclear-encoded form II RuBisCO in dinoflagellates. Science. 268:1622–1624. 10.1126/science.7777861. 7777861

[kiag507-B65] Nam O et al 2024. A protein blueprint of the diatom CO_2_-fixing organelle. Cell. 187:5935–5950.e18. 10.1016/j.cell.2024.09.025. 39368476

[kiag507-B66] Oh ZG et al 2023. A linker protein from a red-type pyrenoid phase separates with rubisco via oligomerizing sticker motifs. Proc Natl Acad Sci U S A. 120:e2304833120. 10.1073/pnas.2304833120. 37311001 PMC10288592

[kiag507-B67] Palmqvist K, Ögren E, Lernmark U. 1994. The CO_2_-concentrating mechanism is absent in the green alga *Coccomyxa*: a comparative study of photosynthetic CO_2_ and light responses of *Coccomyxa*, *Chlamydomonas reinhardtii* and barley protoplasts. Plant Cell Environ. 17:65–72. 10.1111/j.1365-3040.1994.tb00266.x.

[kiag507-B68] Perez FF, Fraga F. 1987. Association constant of fluoride and hydrogen ions in seawater. Mar Chem. 21:161–168. 10.1016/0304-4203(87)90036-3.

[kiag507-B69] Price G, Badger M. 1985. Inhibition by proton buffers of photosynthetic utilization of bicarbonate in *Chara corallina*. Aust J Plant Physiol. 12:257–267. 10.1071/PP9850257.

[kiag507-B70] Rae BD, Long BM, Badger MR, Price GD. 2013. Functions, compositions, and evolution of the two types of carboxysomes: polyhedral microcompartments that facilitate CO_2_ fixation in cyanobacteria and some proteobacteria. Microbiol Mol Biol Rev. 77:357–379. 10.1128/MMBR.00061-12. 24006469 PMC3811607

[kiag507-B71] Raven JA, Beardall J. 2016. The ins and outs of CO_2_. J Exp Bot. 67:1–13. 10.1093/jxb/erv451. 26466660 PMC4682431

[kiag507-B72] Raven JA, Beardall J, Giordano M. 2014. Energy costs of carbon dioxide concentrating mechanisms in aquatic organisms. Photosynth Res. 121:111–124. 10.1007/s11120-013-9962-7. 24390639

[kiag507-B73] Raven JA, Beardall J, Sánchez-Baracaldo P. 2017. The possible evolution and future of CO_2_-concentrating mechanisms. J Exp Bot. 68:3701–3716. 10.1093/jxb/erx110. 28505361

[kiag507-B74] Raven JA et al 2002. Mechanistic interpretation of carbon isotope discrimination by marine macroalgae and seagrasses. Aust J Plant Physiol. 29:355–378. 10.1071/PP01201. 32689482

[kiag507-B75] Raven JA, Suggett DJ, Giordano M. 2020. Inorganic carbon concentrating mechanisms in free-living and symbiotic dinoflagellates and chromerids. J Phycol. 56:1377–1397. 10.1111/jpy.13050. 32654150

[kiag507-B76] Raymond PA et al 2013. Global carbon dioxide emissions from inland waters. Nature. 503:355–359. 10.1038/nature12760. 24256802

[kiag507-B77] Reinfelder JR . 2011. Carbon concentrating mechanisms in eukaryotic marine phytoplankton. Annu Rev Mar Sci. 3:291–315. 10.1146/annurev-marine-120709-142720. 21329207

[kiag507-B78] Reinfelder JR, Kraepiel AM, Morel FM. 2000. Unicellular C_4_ photosynthesis in a marine diatom. Nature. 407:996–999. 10.1038/35039612. 11069177

[kiag507-B79] Reiskind JB, Bowes G. 1991. The role of phosphoenolpyruvate carboxykinase in a marine macroalga with C_4_-like photosynthetic characteristics. Proc Natl Acad Sci U S A. 88:2883–2887. 10.1073/pnas.88.7.2883. 11607173 PMC51344

[kiag507-B80] Rubio L et al 2017. Direct uptake of HCO_3_^−^ in the marine angiosperm *Posidonia oceanica* (L.) Delile driven by a plasma membrane H^+^ economy. Plant Cell Environ. 40:2820–2830. 10.1111/pce.13057. 28815648

[kiag507-B81] Rost B, Riebesell U, Burkhardt S. 2003. Sültemeyer D. Carbon acquisition of bloom-forming marine phytoplankton. Limnol Oceanogr. 48:55–67. 10.4319/lo.2003.48.1.0055.

[kiag507-B82] Sage RF . 2004. The evolution of C_4_ photosynthesis. New Phytol. 161:341–370. 10.1111/j.1469-8137.2004.00974.x. 33873498

[kiag507-B83] Sharwood RE, Ghannoum O, Kapralov MV, Gunn LH, Whitney SM. 2016. Temperature responses of Rubisco from Paniceae grasses provide opportunities for improving C_3_ photosynthesis. Nat Plants. 2:16186. 10.1038/nplants.2016.186. 27892943

[kiag507-B84] Shimakawa G et al 2024. Diatom pyrenoids are encased in a protein shell that enables efficient CO_2_ fixation. Cell. 187:5919–5934.e19. 10.1016/j.cell.2024.09.013. 39357521

[kiag507-B85] Sobek S, Tranvik LJ, Prairie YT, Kortelainen P, Cole JJ. 2007. Patterns and regulation of dissolved organic carbon: an analysis of 7,500 widely distributed lakes. Limnol Oceanogr. 52:1208–1219. 10.4319/lo.2007.52.3.1208.

[kiag507-B86] Sobrino C, Ward ML, Neale PJ. 2008. Acclimation to elevated carbon dioxide and ultraviolet radiation in the diatom *Thalassiosira pseudonana*: effects on growth, photosynthesis, and spectral sensitivity of photoinhibition. Limnol Oceanogr. 53:494–505. 10.4319/lo.2008.53.2.0494.

[kiag507-B87] Somerville GA, Proctor RA. 2013. Cultivation conditions and the diffusion of oxygen into culture media: the rationale for the flask-to-medium ratio in microbiology. BMC Microbiol. 13:9. 10.1186/1471-2180-13-9. 23324109 PMC3551634

[kiag507-B88] Steensma AK et al 2025. Modeling with uncertainty quantification reveals the essentials of a non-canonical algal carbon-concentrating mechanism. Plant Physiol. 197:kiae629. 10.1093/plphys/kiae629. 39656810 PMC11836721

[kiag507-B89] Tcherkez GG, Bathellier C, Farquhar GD, Lorimer GH. 2018. Commentary: directions for optimization of photosynthetic carbon fixation: RuBisCO's efficiency may not be so constrained after all. Front Plant Sci. 9:929. 10.3389/fpls.2018.00929. 29997647 PMC6030380

[kiag507-B90] Tcherkez GG, Farquhar GD. 2021. Rubisco catalytic adaptation is mostly driven by photosynthetic conditions—not by phylogenetic constraints. J Plant Physiol. 267:153554. 10.1016/j.jplph.2021.153554. 34749030

[kiag507-B91] Tcherkez GG, Farquhar GD, Andrews TJ. 2006. Despite slow catalysis and confused substrate specificity, all ribulose bisphosphate carboxylases may be nearly perfectly optimized. Proc Natl Acad Sci U S A. 103:7246–7251. 10.1073/pnas.0600605103. 16641091 PMC1464328

[kiag507-B92] Tortell PD et al 2008. Inorganic carbon uptake by Southern Ocean phytoplankton. Limnol Oceanogr. 53:1266–1278. 10.4319/lo.2008.53.4.1266.

[kiag507-B93] van der Loos LM et al 2019. Responses of macroalgae to CO_2_ enrichment cannot be inferred solely from their inorganic carbon uptake strategy. Ecol Evol. 9:125–140. 10.1002/ece3.4679. 30680101 PMC6342131

[kiag507-B94] Whitehead L, Long BM, Price GD, Badger MR. 2014. Comparing the in vivo function of α-carboxysomes and β-carboxysomes in two model cyanobacteria. Plant Physiol. 165:398–411. 10.1104/pp.114.237941. 24642960 PMC4012598

[kiag507-B95] Whitney SM, Andrews TJ. 1998. The CO_2_/O_2_ specificity of single-subunit ribulose-bisphosphate carboxylase from the dinoflagellate, *Amphidinium carterae*. Aust J Plant Physiol. 25:131–138. 10.1071/PP97131.

[kiag507-B96] Yeoh H-H, Badger MR, Watson L. 1981. Variations in kinetic properties of ribulose-1,5-bisphosphate carboxylases among plants. Plant Physiol. 67:1151–1155. 10.1104/pp.67.6.1151. 16661826 PMC425851

[kiag507-B97] Young JN et al 2016. Large variation in the Rubisco kinetics of diatoms reveals diversity among their carbon-concentrating mechanisms. J Exp Bot. 67:3445–3456. 10.1093/jxb/erw163. 27129950 PMC4892730

[kiag507-B98] Zeebe RE . 2011. On the molecular diffusion coefficients of dissolved CO_2_, HCO_3_^−^, and CO_3_^2−^ and their dependence on isotopic mass. Geochim Cosmochim Acta. 75:2483–2498. 10.1016/j.gca.2011.02.010.

[kiag507-B99] Zhou R-Q et al 2024. Structure and assembly of the α-carboxysome in the marine cyanobacterium *Prochlorococcus*. Nat Plants. 10:661–672. 10.1038/s41477-024-01660-9. 38589484

